# FtsE, the Nucleotide Binding Domain of the ABC Transporter Homolog FtsEX, Regulates Septal PG Synthesis in *E. coli*

**DOI:** 10.1128/spectrum.02863-22

**Published:** 2023-04-04

**Authors:** Sunanda Mallik, Hiren Dodia, Arup Ghosh, Ramanujam Srinivasan, Liam Good, Sunil Kumar Raghav, Tushar Kant Beuria

**Affiliations:** a Institute of Life Sciences, Nalco Square, Bhubaneswar, Odisha, India; b Manipal Academy of Higher Education, Manipal, Karnataka, India; c Regional Centre for Biotechnology, Faridabad, Haryana, India; d National Institute of Science Education and Research, Bhubaneswar, Odisha, India; e The Royal Veterinary College, University of London, London, United Kingdom; Centre National de la Recherche Scientifique, Aix-Marseille Université

**Keywords:** FtsE, FtsX, FtsZ, PG hydrolysis, PG synthesis, divisome complex

## Abstract

The peptidoglycan (PG) layer, a crucial component of the tripartite *E.coli* envelope, is required to maintain cellular integrity, protecting the cells from mechanical stress resulting from intracellular turgor pressure. Thus, coordinating synthesis and hydrolysis of PG during cell division (septal PG) is crucial for bacteria. The FtsEX complex directs septal PG hydrolysis through the activation of amidases; however, the mechanism and regulation of septal PG synthesis are unclear. In addition, how septal PG synthesis and hydrolysis are coordinated has remained unclear. Here, we have shown that overexpression of FtsE leads to a mid-cell bulging phenotype in *E.coli*, which is different from the filamentous phenotype observed during overexpression of other cell division proteins. Silencing of the common PG synthesis genes *murA* and *murB* reduced bulging, confirming that this phenotype is due to excess PG synthesis. We further demonstrated that septal PG synthesis is independent of FtsE ATPase activity and FtsX. These observations and previous results suggest that FtsEX plays a role during septal PG hydrolysis, whereas FtsE alone coordinates septal PG synthesis. Overall, our study findings support a model in which FtsE plays a role in coordinating septal PG synthesis with bacterial cell division.

**IMPORTANCE** The peptidoglycan (PG) layer is an essential component of the *E.coli* envelope that is required to maintain cellular shape and integrity. Thus, coordinating PG synthesis and hydrolysis at the mid-cell (septal PG) is crucial during bacterial division. The FtsEX complex directs septal PG hydrolysis through the activation of amidases; however, its role in regulation of septal PG synthesis is unclear. Here, we demonstrate that overexpression of FtsE in *E.coli* leads to a mid-cell bulging phenotype due to excess PG synthesis. This phenotype was reduced upon silencing of common PG synthesis genes *murA* and *murB*. We further demonstrated that septal PG synthesis is independent of FtsE ATPase activity and FtsX. These observations suggest that the FtsEX complex plays a role during septal PG hydrolysis, whereas FtsE alone coordinates septal PG synthesis. Our study indicates that FtsE plays a role in coordinating septal PG synthesis with bacterial cell division.

## INTRODUCTION

Cell division in Escherichia coli is an orchestrated process which occurs through a constrictive mode (1). The division process initiates at the mid-cell with the assembly of tubulin-like protein FtsZ, which forms a ring-like structure called the Z-ring (2, 3). The formation of the Z-ring is followed by the sequential recruitment of several essential and nonessential proteins to the division site, creating a dynamic complex known as the divisome (4). The component proteins of the divisome complex are involved in several crucial processes such as maintaining the dynamic nature of the Z-ring (5), anchoring the Z-ring to the inner membrane (4, 6), coordinating cell division with nucleoid segregation (7), coordinating septal cell wall hydrolysis and synthesis, etc. (8). In E. coli, the cell wall consists of a peptidoglycan (PG) layer positioned between the inner and outer membrane and is an essential protective boundary for bacteria to maintain shape and turgor pressure; therefore, the division of all three layers must be tightly coordinated (9). The inner membrane invagination during cell division depends on the force generated by FtsZ constriction (10) and the outer membrane invagination depends upon septal PG synthesis (11). Also, recent reports have suggested that the inner membrane invagination (12) and septal PG synthesis synchronize with the treadmilling properties of FtsZ (13, 14).

In *E.coli*, septal PG hydrolysis is partially controlled by the FtsEX protein complex, which is a homolog of the ATP binding cassette (ABC) transporter (type VII family) (15, 16). In the cytoplasm, FtsE hydrolyzes ATP and cause a structural change in periplasmic protein EnvC through FtsX (17–19). This event leads to the activation of amidases A and B (17), which in turn initiates PG hydrolysis at the mid-cell. More recently, FtsX was shown to interact with FtsA for the recruitment of late-cell division proteins (20), which suggests that besides PG hydrolysis, FtsX also performs a role in divisome assembly. Similarly, FtsE, the cytoplasmic domain of the FtsEX complex, localizes to the mid-cell through its interaction with the C-terminal core domain of FtsZ, an interaction that is independent of FtsX (2, 21). Most divisome regulatory proteins, including MinC, SlmA, and MinD, bind to the core domain of FtsZ and thus regulate its function to precisely localize at the mid-cell (22–25). The interaction of FtsE with FtsZ follows a pattern similar to the aforementioned proteins, i.e., FtsE interacts with the core domain of FtsZ, which suggests that FtsE may also play a regulatory role during cell division. Additionally, the daughter cell separation requires perfect coordination between cell constriction and septal cell wall hydrolysis and synthesis. The interaction of FtsE with FtsZ in the cytoplasm and that of FtsX with EnvC in the periplasm during bacterial division are probably coordinated processes. In this study, we have discovered the independent role of FtsE during bacterial division. Our study shows that overexpression of FtsE leads to higher septal PG synthesis, which is independent of FtsX, and suggests that while FtsE regulates PG synthesis, the FtsEX complex regulates PG hydrolysis during bacterial division.

## RESULTS

### Overexpression of FtsE in *E. coli* does not influence bacterial division.

**FIG 1:**
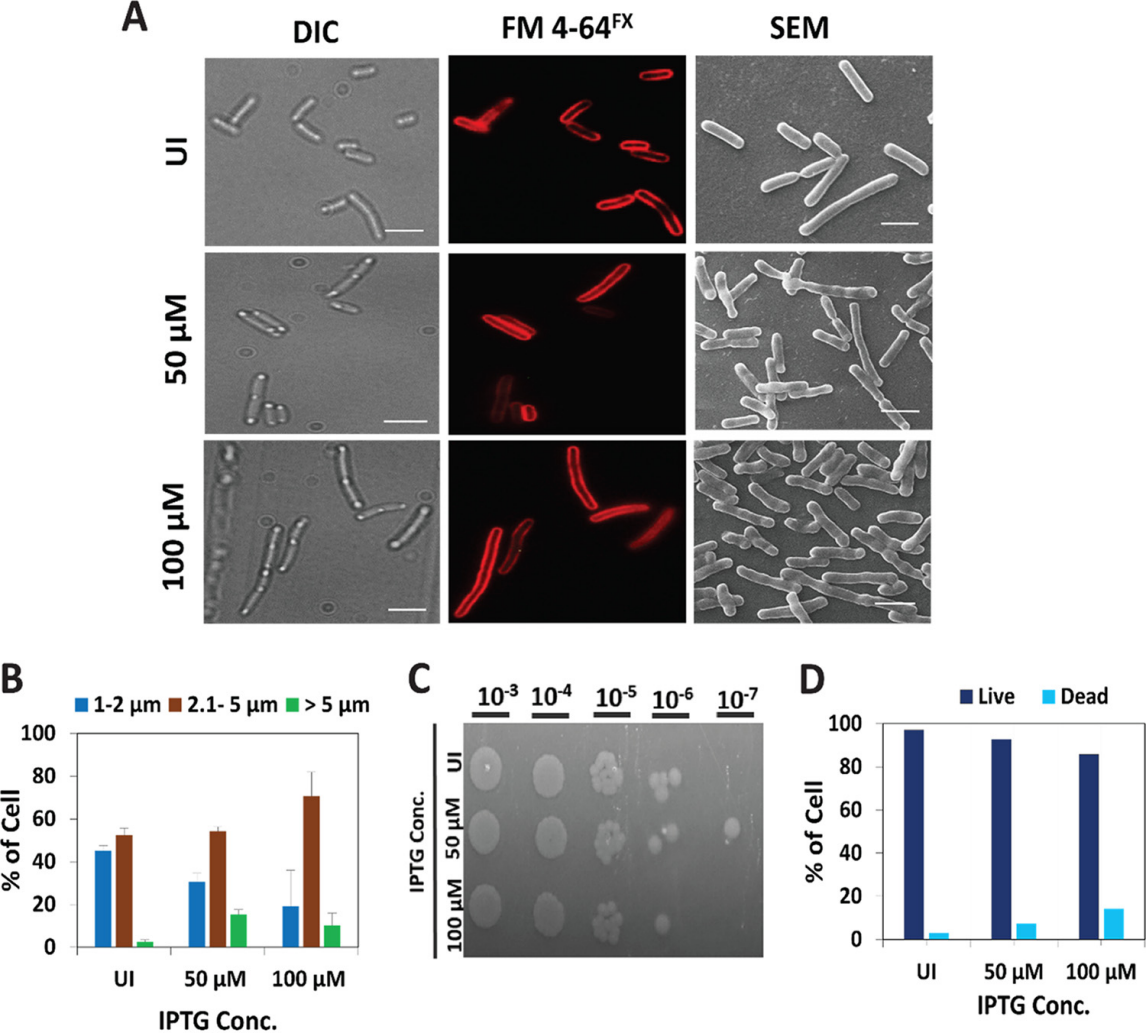
Overexpression of FtsE shows bulging morphology. (A) Differential Interference Contrast (DIC) images, inner membrane (FM 4 64^FX^, red) and Scanning electron microscope images of Escherichia coli under uninduced (UI) or different FtsE overexpression (50 and 100 μM IPTG [isopropyl β-d-thiogalactopyranoside]) conditions. Scale bar = 4 μm. (B to D) Lengths of bacteria (B), cell viability (C), and percentage of live-dead bacteria (D) when FtsE is overexpressed.

To explore whether, like FtsX, FtsE also executes any independent function during cell division, we assessed the morphological changes in FtsE-overexpressing *E.coli* cells (MG1655). Unlike other division proteins, overexpression of FtsE did not form filamentous cells, instead showed a unique bulging morphology at both mid-cell and the poles (Fig. 1A; Fig. S1B and Fig. S2C in the supplemental material). The bulging morphology started to appear in the presence of as low as 25 μM IPTG (isopropyl β-d-thiogalactopyranoside) in few cells; however, at 50-μM or higher concentrations, almost all cells showed the bulging morphology. Because the FtsEX complex is involved in septal PG hydrolysis, we initially presumed that overexpression of FtsE would lead to excessive PG hydrolysis, which in turn would lead to the bulging morphology. To corroborate this, the inner membrane structure was visualized by staining with FM 4-64^FX^ and found to be intact (Fig. 1A), suggesting no cell death during FtsE overexpression. Moreover, our dilution plating results confirmed that there was no significant cell death upon FtsE overexpression (Fig. 1C). Cell death was also investigated using a Live-Dead assay and was found to be nonsignificant (Fig. 1D; Fig. S2A). However, under the overexpression condition, the dividing cells were found to be longer than the control cells (Fig. 1B).

**FIG 2:**
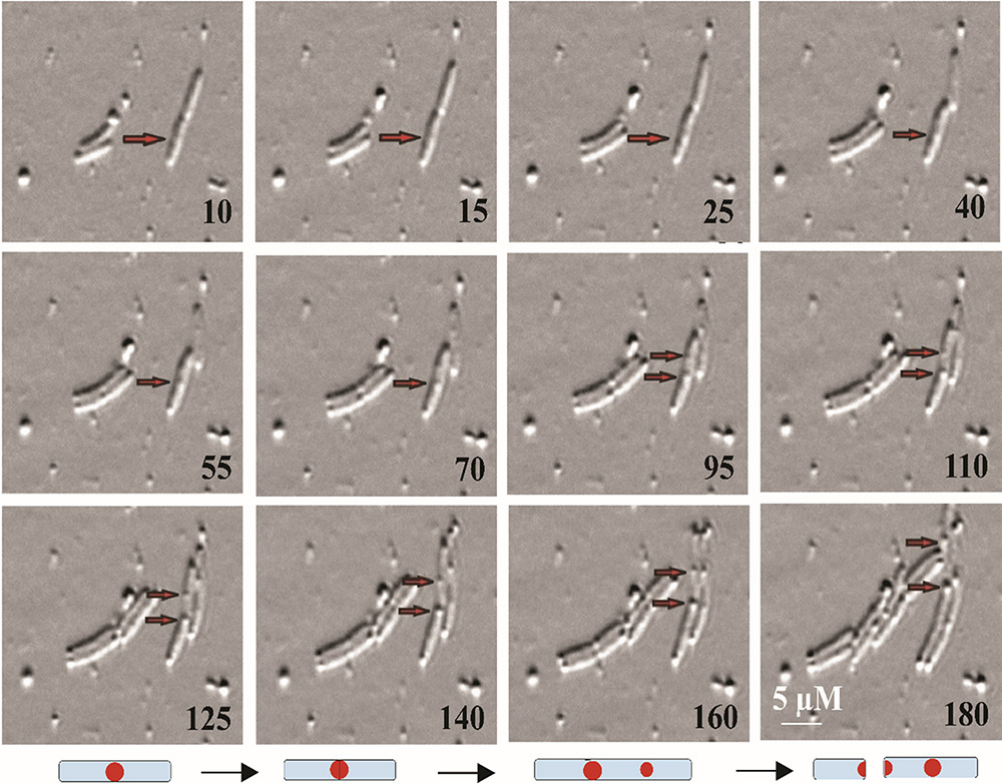
Bulging initiates at the mid-cell. IPTG was used to induce FtsE. After 1 h of FtsE induction, cells were observed under a microscope. Time-lapse images show movement of the bulges from mid-cell in the dividing bacterium to the poles in the daughter cells.

Furthermore, to confirm that the bulging morphology is purely a result of FtsE overexpression, we overexpressed other proteins (FtsZ, MinD, SulA, and SlmA) involved in E. coli division using the same expression system. Overproduction of these division proteins leads to cell filamentation rather than any bulging morphology (Fig. S1A). All these observations suggest that the bulging morphology is a specific morphological change caused by FtsE overexpression. The absence of filamented cells upon FtsE overexpression also reveals that FtsE overexpression does not inhibit divisome assembly. The bulging morphology upon FtsE overexpression was observed at both mid-cell and the poles. To confirm that the bulging morphology is not a random process and is specific at the mid-cell, resulting in bulges appearing at the poles of the newborn cell, we examined the origin and site of bulging by performing a time-lapse experiment. It appeared that the bulging began at the mid-cell and then moved to the poles after subsequent rounds of cell division (Fig. 2; supplemental material file S1). As a consequence of this, the daughter cells showed bulges at both mid-cell and the poles. Through immunostaining, we also checked the localization of FtsE to the bulging site (Fig. S1B). Consistent with all these observations, we concluded that bulging upon FtsE overexpression is a specific morphological change which initiates at the mid-cell and then migrates to the new poles (Fig. 2; SV1).

### Excess PG synthesis leads to bulging morphology in FtsE overexpressed cells.

**FIG 3:**
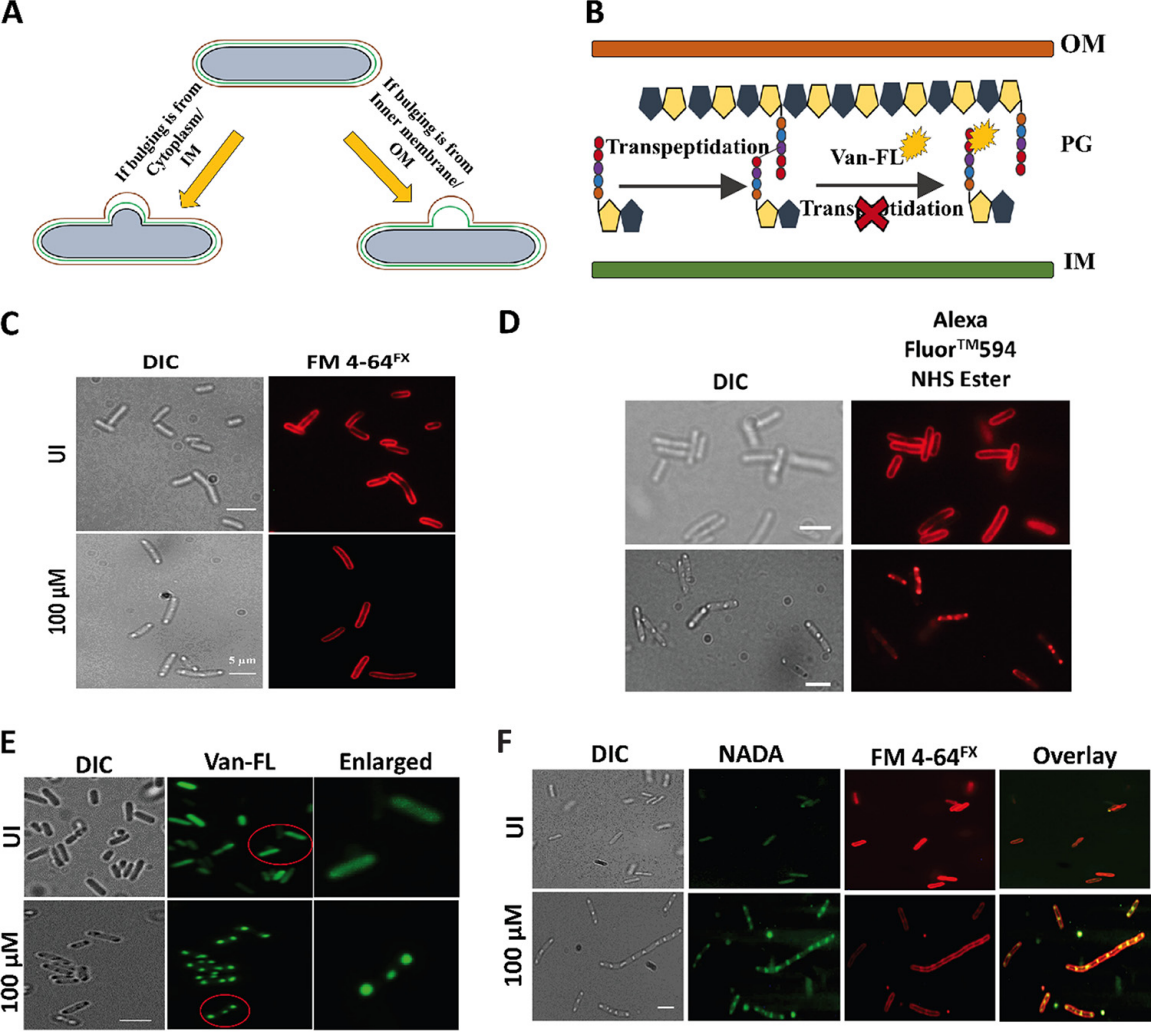
Overexpression of FtsE causes deformation in the outer membrane of E. coli. (A) Cartoon showing peptidoglycan (PG) layer in E. coli and how the cell would appear if the bulging initiates at the cytoplasm, inner membrane, or outer membrane. (B) Cartoon showing fluorescent vancomycin labeling. (C) An intact inner membrane (FM 4-64^FX^) during FtsE overexpression. (D) Excessive staining with Alexa Fluor 594 NHS Ester. (E) Excessive staining at bulging site with fluorescent vancomycin (Van-FL). (F) Live staining with the fluorescence analog of d-alanine (NADA). Scale bar = 4 μm.

The bulging morphology could result from reactions occurring in the cytoplasm or from membrane deformation. To better understanding the causes of the bulging morphology, we labeled the outer envelope with a fluorescent dye and observed it under a fluorescence microscope. A cartoon representation in Fig. 3A shows the cell morphology when bulging was initiated at different layers. FM 4-64^FX^ (26) was used to stain the inner membrane, Alexa Fluor 594 NHS Ester was used to stain the outer membrane (27, 28), and fluorescent vancomycin (Van-FL) (29) and a D-Ala fluorescent probes (NADA) (30) were used to stain the PG layers. The FM 4-64^FX^ staining clearly showed the presence of an intact inner membrane, suggesting that the bulging phenotype was not a cytoplasmic or inner membrane event (Fig. 3C). Cell staining with Alexa Fluor 594 NHS Ester, a hydrophilic compound which labels the primary amines of proteins and other amine-containing molecules in the outer membrane, showed intense staining in the bulging region. In contrast, very faint staining was observed in other parts of the cell (Fig. 3D). Since the peptidoglycan layer lies outside the inner membrane and FtsE is involved in PG hydrolysis, we confirmed the involvement of the PG layer in triggering the bulging morphology. If the bulging had been a consequence of excessive PG hydrolysis, we would have seen cell death due to cell lysis; however, no significant death was observed during any of our experiments (Fig. 1C; Fig. S2A). In contrast, our experiments revealed the presence of new PG precursors, showing increased PG synthesis at the bulging site. We used fluorescent Van-FL to stain the nascent PG. Van-FL binds only to PG precursors, which contain two d-alanines in the short pentapeptide (31). However, in the older PG precursor, the d-alanine at the fourth position is used for transpeptidation after the removal of the d-alanine at the fifth position. Due to the absence of the last d-alanine in the pentapeptide, vancomycin cannot bind to the older PG precursors; however, it can bind to newly synthesized PG precursors in which transpeptidation has not yet occurred (Fig. 3B). Van-FL labeling showed a strong fluorescence signal at the bulging region, indicating that the bulging morphology is a result of excessive PG synthesis (Fig. 3E). The result was further supported with NADA labeling: NADA is a fluorescent analogue of d-alanine and, when added to medium during bacterial growth, is taken up by bacteria and incorporated into the PG layer. We found that the pattern of NADA labeling was similar to that of the Van-FL labeling (Fig. 3F). These findings together confirmed that the bulging morphology triggered by FtsE overexpression was due to excess peptidoglycan synthesis (Fig. 3).

### FtsE overexpression activates the PG synthesis pathway in *E. coli*.

**FIG 4:**
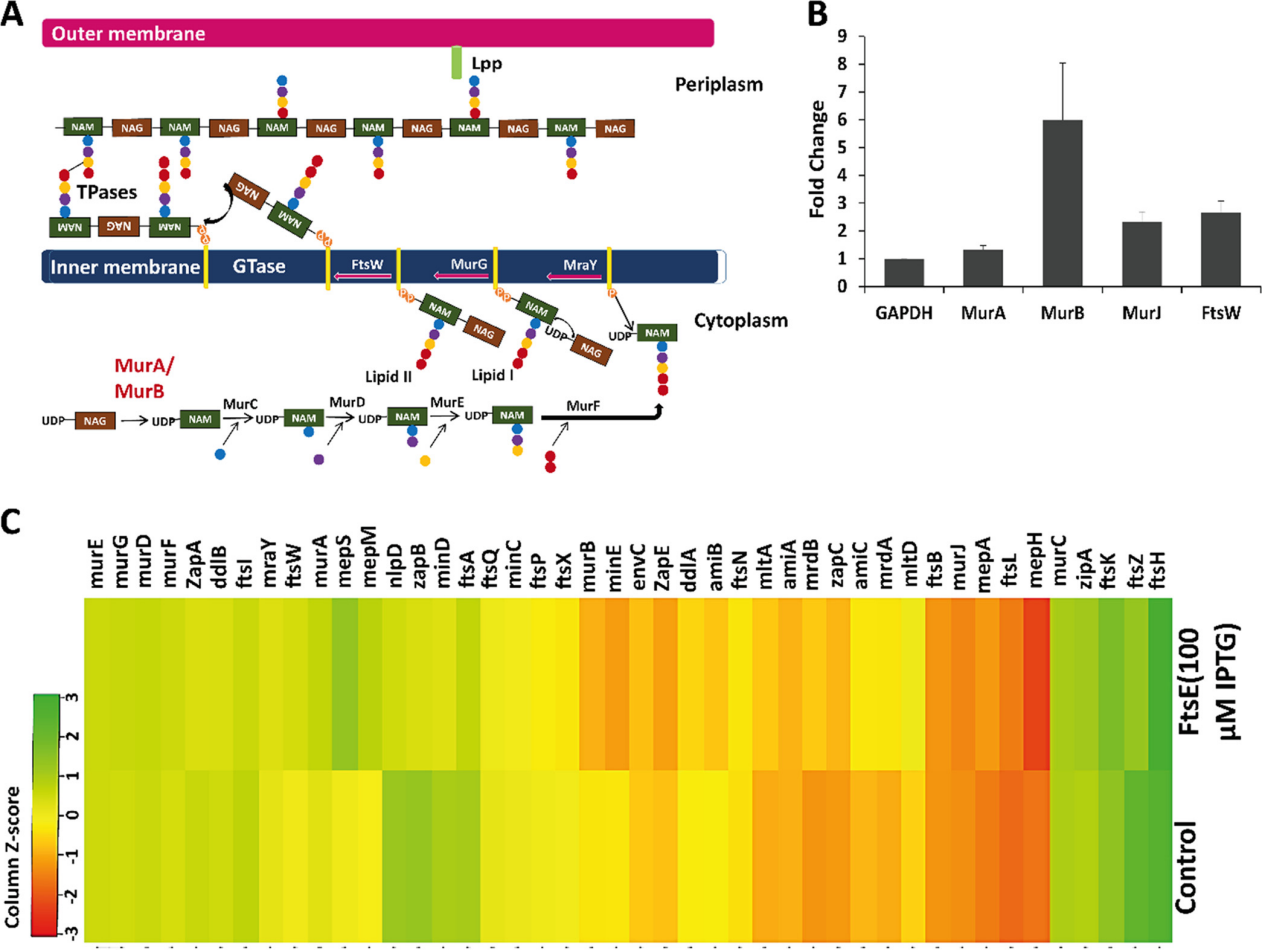
Transcriptome profiling of FtsE-overexpressing cell (MG1655). (A) Cartoon showing the septal PG synthesis pathway in *E. coli*. (B) Quantitative real-time PCR data for *murA* and *murB* (involved in PG synthesis) and *ftsW* and *murJ* (flippase) showing the fold changes in gene expression observed during FtsE overexpression. Data were normalized with GAPDH (glyceraldehyde-3-phosphate dehydrogenase). (C) Transcriptome profiling of E. coli MG1655 cells overexpressing FtsE.

PG synthesis in E. coli involves three steps: (i) the cytoplasmic step, where the new PG precursors are synthesized; (ii) the inner membrane step, where the PG precursors are anchored to the inner membrane through a lipid II molecule and flipped to the periplasmic side with the help of a flippase; and (iii) the periplasmic step, where the newly flipped PG precursors are inserted into the old PG strand through transglycosylation followed by transpeptidation (Fig. 4A) (32). To determine which step of the PG synthesis pathway is activated during FtsE overexpression, we monitored changes in the expression levels of genes involved at each step during FtsE overexpression (Fig. 4A). In addition to PG synthesis events, we also checked genes involved in cell division and PG hydrolysis using Real-Time Quantitative Reverse Transcription PCR (qRT-PCR). Interestingly, from qRT-PCR, we observed that the mRNA levels of most PG synthesis genes increased during FtsE overexpression. For example, we found a 1.5- to 2-fold increase in the mRNA levels of almost all PG synthesis genes except for *murB* (Fig. 4B; Fig. S3A). The result was further supported by transcriptome analysis, where we observed increased expression of genes involved in the PG synthesis pathway (Fig. 4C, Fig. S3B). From the combined qRT-PCR and transcriptome data, we found an increase in the mRNA level of almost all PG synthesis genes, including *murA*, *murB*, and *ftsW* (Fig. 4B and C; Fig. S3A to B). Overall, the qRT-PCR and transcriptome data supported our assumption that FtsE overexpression activates septal PG synthesis (Fig. 4; Fig. S3). Apart from the increased expression of PG synthesis genes, we also observed increased expression of PG hydrolysis genes using both methods. Interestingly, we also observed an increase in the expression levels of several genes involved in bacterial division, including *ftsK*, *ftsQ*, *ftsL*, *ftsB*, and *ftsN*. Surprisingly, we did not observe any increase in the expression level of *ftsI*, a transpeptidase responsible for the incorporation of newly synthesized PG precursors at the septum.

### Silencing of *murA* and *murB* genes confirm the bulging morphology is a consequence of PG synthesis.

**FIG 5:**
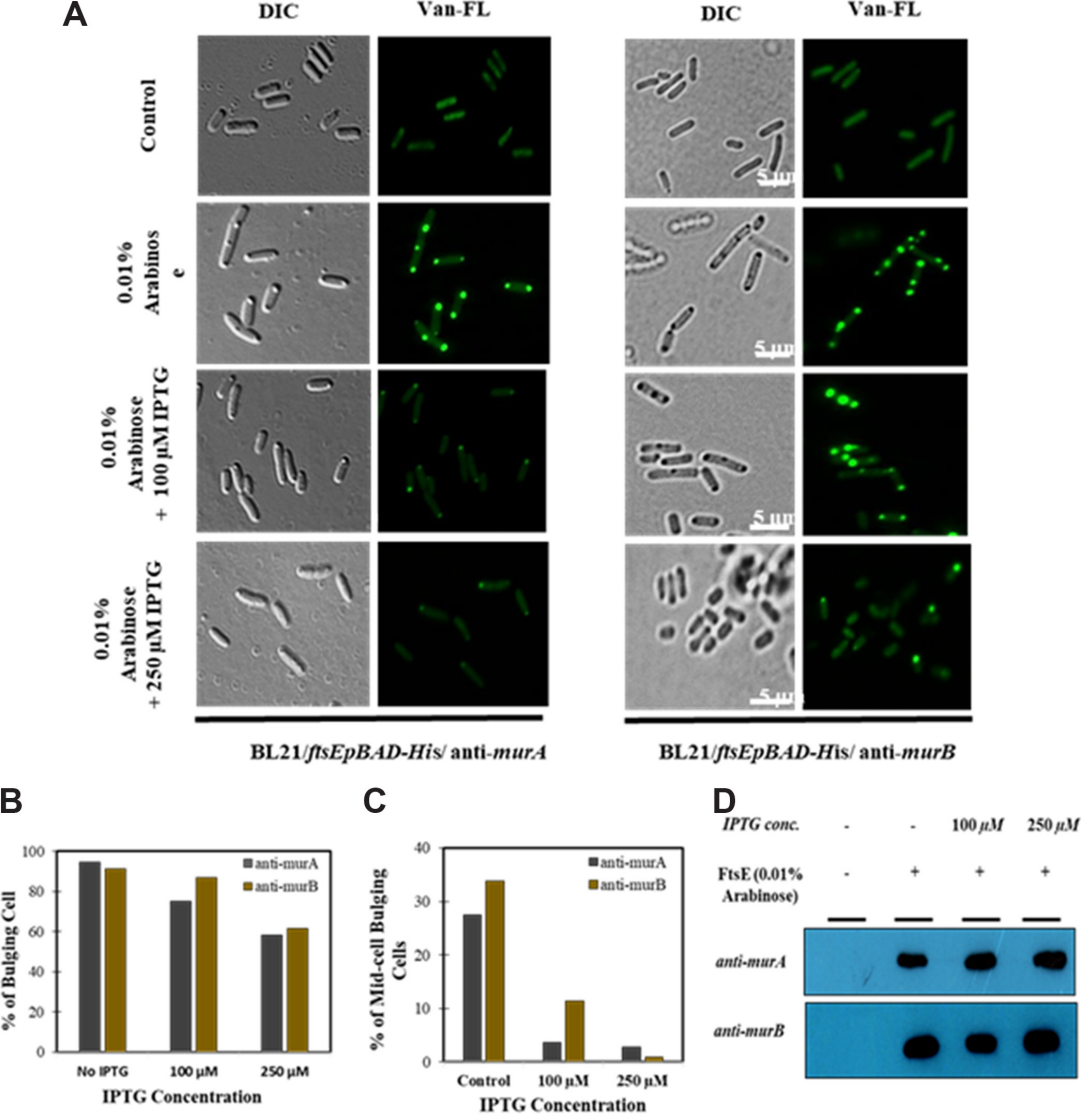
Silencing of *murA* and *murB* through antisense RNA in FtsE-overexpressing E. coli. Bulging morphology in E. coli was induced by overexpressing FtsE using arabinose (0.01%). The antisense RNAs against MurA or MurB were expressed using IPTG. (A) Reduction in bulging morphology upon *murA* (left) and *murB* (right) silencing. Fluorescent vancomycin (Van-FL) labeling was also performed to confirm this reduction. (B) Percentage of cells with bulging phenotype, where antisense *murA* and *murB* were expressed using 100 and 250 μM IPTG. The numbers of cells counted in each case are as follows: *murA* control (*n* = 392), *anti-murA* 100 μM IPTG (*n* = 216), and *anti-murA* 250 μM IPTG (*n* = 284); and *murB* control (*n* = 381), *anti-murB* 100 μM IPTG (*n* = 432), and *anti-murB* 250 μM IPTG (*n* = 331). (C) Percentage of cells with only mid-cell bulging. (D) Similar levels of FtsE protein observed when *murA* and *murB* were silenced. Scale bar = 5 μm.

Our earlier experiments showed that FtsE overexpression in E. coli increased the expression of PG synthesis genes at the mid-cell, resulting in bulging morphology. If this is true, then the knockdown of PG synthesis genes should influence the bulging morphology. Since MurA and MurB are essential enzymes involved in the initial steps of the PG synthesis pathway (Fig. 4A), inhibition of *murA* and *murB* at the mRNA level can be used to confirm the involvement of PG synthesis in inducing bulging morphology. To do this, we used anti-sense RNA technology, where the antisense RNA was expressed using IPTG (33). Because the antisense RNA requires IPTG to be expressed, FtsE was cloned under an arabinose promoter (18). Interestingly, with increasing antisense expression against either *murA* or *murB*, the bulging morphology in E. coli decreased considerably (Fig. 5A and B). Previously, we showed that the bulging morphology was initiated at the mid-cell. Interestingly, upon *murA* or *murB* silencing, mid-cell bulging morphology was decreased in almost all cells, suggesting that inhibition of PG synthesis prevents the initiation of mid-cell bulging (Fig. 5C). To rule out the possibility that the reduced bulging during silencing was not a result of low FtsE expression, we performed a Western blot analysis and found that an equal amount of FtsE protein was present under both the control and silenced conditions. (Fig. 5D). We also determined cell viability upon silencing and found no significant decrease in viable cells even at higher concentrations of IPTG (Fig. S4). Together, our results indicated that FtsE may play a role during septal PG synthesis in E. coli.

Our qRT-PCR and transcriptome analysis also showed increases in the expression levels of PG polymerase and flippases such as *ftsW* and *murJ* (Fig. 4, Fig. S3). These two enzymes together insert newly synthesized PG precursors from the cytoplasmic site to the old PG strand during PG synthesis; therefore, we examined their involvement in bulging morphology. Surprisingly, compared to depletion of *murA* and *murB*, depletion of *ftsW* and *murJ* during FtsE overexpression did not affect the bulging morphology (Fig. S5A and B). Similarly, we also observed no change in FtsE expression levels during the downregulation of *ftsW* and *murJ* (Fig. S5C).

### Mid-cell localization of FtsE is crucial for septal PG synthesis.

**FIG 6:**
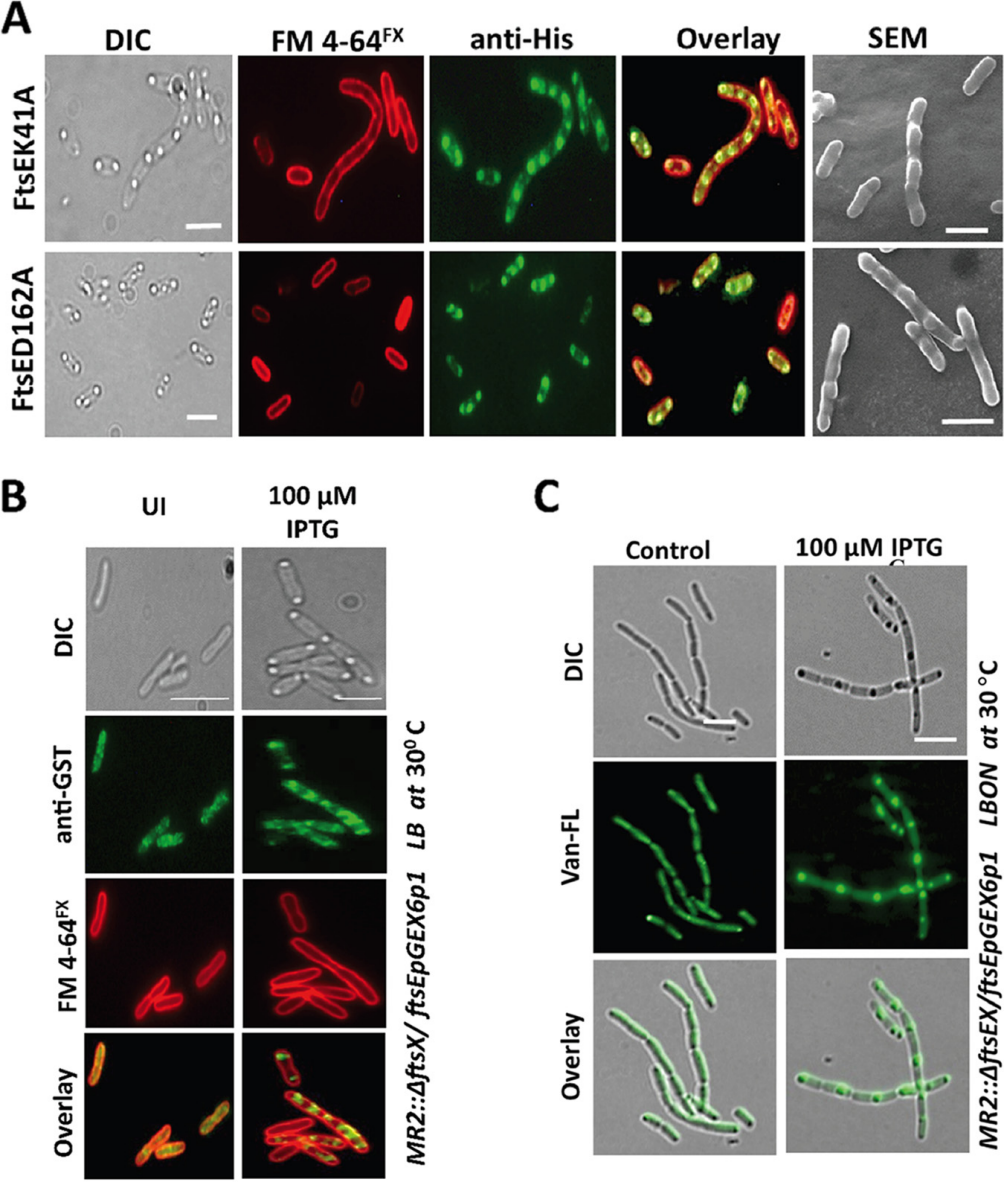
The mid-cell localization, but not the ATPase activity, of FtsE is necessary for PG synthesis in E. coli, and the activation of PG synthesis by FtsE is independent of FtsX and PG hydrolysis. (A) DIC, fluorescence microscopy, and scanning electron microscopy (SEM) images of E. coli overexpressing different FtsE mutants. Scale bars = 4 μm and 2 μm (SEM). (B) FtsE localizes to the bulging site and mid-cell in the absence of FtsX (scale bar = 4 μm). (C) Septal PG synthesis occurs even in a FtsX deletion strain, which forms a chaining phenotype due to inhibition of septal PG hydrolysis (scale bar = 5 μm).

An ATPase mutant of FtsE (D162N) did not compensate for the PG hydrolysis-deficient phenotype even in the presence of FtsX, suggesting that the ATPase activity of FtsE is necessary for PG hydrolysis (17). We also wanted to determine the involvement of FtsE ATPase activity in septal PG synthesis. To assess this, we generated FtsE ATPase mutants deficient in either ATP binding (K41A in P-loop) or ATP hydrolysis (D162A in D-loop) (34). Both FtsE mutants (FtsE^K41A^ and FtsE^D162A^), when overexpressed, exhibited the bulging morphology (Fig. 6). SDS-PAGE analysis showed that similar levels of FtsE protein were expressed in these mutants (Fig. S8A). Like wild-type FtsE, both FtsE^K41A^ and FtsE^D162A^ were able to localize to the mid-cell bulging (Fig. 6A). We also observed that overexpression of FtsE^D162A^ is more toxic than overexpression of FtsE^K41A^ (Fig. S6). From these results, we infer that neither the ATP binding nor the ATPase activity of FtsE are necessary for the initiation of the bulging morphology.

### Septal PG synthesis is independent of septal PG hydrolysis and FtsX.

As with ATPase mutants of FtsE, deletion of the extra periplasmic loop of FtsX does not rescue the chaining phenotype, suggesting that FtsX is importance during septal PG hydrolysis (17). Our results indicated that FtsE is involved in the activation of septal PG synthesis. Next, we wanted to examine whether the whole FtsEX complex or FtsE alone can activate septal PG synthesis. We overexpressed FtsE in a FtsX deletion background and checked for the bulging morphology. In the absence of FtsX, we observed bulging morphology, suggesting that unlike for septal PG hydrolysis, FtsE does not require FtsX for septal PG synthesis (Fig. S7A). Since FtsE can generate a bulging morphology in the absence of FtsX and interacts with FtsZ (21), then FtsE should localize to the mid-cell even in the absence of FtsX. Immunofluorescence microscopy was performed to further visualize the localization of FtsE at the bulging site in the absence of FtsX. We observed that the cells with bulges displayed mid-cell localization of FtsE in the FtsX deletion background (Fig. 6B). Thus, it appears that FtsE alone is sufficient to initiate septal PG synthesis during E. coli division. If initiation of septal PG synthesis by FtsE is independent of FtsX, it is possible that septal PG synthesis is also independent of septal PG hydrolysis. To validate this, we used a FtsEX deletion strain and overexpressed FtsE alone in LBON medium (LB medium without NaCl) (35) at 30°C. Overexpression of FtsE alone in FtsEX deletion strain cannot rescue the chaining phenotype (absence of PG hydrolysis) in LBON medium; however, under similar conditions, the bulging morphology (PG synthesis) was manifested (Fig. 6C; Fig. S7B). This finding suggests that septal PG synthesis is independent of septal PG hydrolysis.

## DISCUSSION

### FtsE-mediated activation of septal PG synthesis.

More than a dozen divisome proteins accomplish successful and precise division in E. coli. Most E. coli divisome proteins are investigated in the context of their localization and their role in divisome assembly. However, very few have been examined for their roles in activities other than cell division, and FtsEX is one of them. The function of the FtsEX complex in PG hydrolysis during cell division is well-studied in different bacterial species (16, 35, 36). During cell division, FtsX performs dual functions: divisome assembly through its interaction with FtsA (19), and septal PG hydrolysis (16) through its interaction with EnvC. Similarly, FtsE interacts with FtsZ even without FtsX (20). The interaction and localization of FtsE indicate its importance in activities other than septal PG hydrolysis. Although several studies have previously reported the involvement of FtsEX complex in PG synthesis, its mechanism was still elusive (19). In this study, we assessed the independent functions of FtsE and showed that it plays a direct role in septal PG synthesis during E. coli division. FtsE is known to be involved in septal PG hydrolysis (16). Hence, overexpression of FtsE could cause excess or uncontrolled PG hydrolysis, leading to breaches in the PG layer and, finally, cell lysis. Unexpectedly, our initial finding showed a unique bulging morphology in FtsE-overexpressing cells, which we assumed to be a sign of cell death. Surprisingly, we did not observe any increase in cell death from dilution plating and a live-dead assay (Fig. 1C and D). Instead of bulging, we observed a filamentation morphology with overexpression of other cell division proteins (i.e., FtsZ, SulA, SlmA, and MinD) (Fig. S1). Because these proteins regulate the Z-ring assembly in a concentration-dependent manner, their overexpression leads to imbalanced protein proportions at the mid-cell and causes instability in the divisome. This finding suggests that unlike the FtsX-FtsA interaction (19), FtsE overexpression did not inhibit divisome assembly. If FtsE overexpression does not hamper divisome stability and bulging is not a sign of cell lysis, we assume that the bulging is due to morphological changes. Initially, morphological changes were clearly observed with scanning electron microscopy (SEM) (Fig. 1A) and transmission electron microscopy (TEM) (Fig. S8B). This hypothesis was later confirmed when the different membrane layers were observed in FtsE-overexpressing cells. Fluorescent vancomycin and NADA labeling further revealed that the morphological changes at the mid-cell originated from the middle PG layer. Because vancomycin binds to the d-alanyl-d-alanine of newly flipped PG precursors and not to the old cross-linked PG, the robust staining of bulging with fluorescent vancomycin indicates that this bulging might be due to excess PG synthesis. With this vision, strong NADA labeling to the bulging site also indicated that this excess PG synthesis is the cause of the mid-cell bulging morphology. Also, staining with Alexa Fluor 594 NHS Ester produced a strong signal at the bulging site, indicating the presence of an excess amine group in the same place. In *E. coli*, the pentapeptide of the PG precursors possesses lysine, which can contribute to its amine group for the staining. Excess PG precursor leads to a spare amine group and strong staining signal (Fig. 3D). Although we initially wanted to determine the function of FtsE in septal PG hydrolysis, we observed its involvement in septal PG synthesis. Next, time-lapse microscopy confirmed that the excess PG synthesis occurred at the mid-cell.

Lateral and septal PG synthesis require almost the same enzymes, except for the flippase. During septal PG synthesis, MurJ localizes to the mid-cell with the help of FtsW and lipid II molecule (new PG precursor) and is involved in the flipping of new PG precursor to the periplasm (35). Surprisingly, our transcriptome data showed an increase in the mRNA levels of almost all PG synthesis genes along with *ftsW* and *murJ*; however, no significant change in the expression level of *ftsI* was observed. Fluorescence vancomycin labeling at the bulging site confirmed the presence of a newly flipped PG precursor, which has not undergone transpeptidation. Because there was no increase in the expression level of *ftsI* upon FtsE overexpression, the PG precursor’s insertion rate to the old glycan strand was low compared to the PG precursor synthesis rate, which resulted in the accumulation of the new PG precursors at the periplasm, causing the bulging morphology. We also saw an increase in *murA* and *murB* mRNA levels; both of these are common enzymes required during the initial steps of the PG synthesis pathway. The upregulation of *ftsW* and *murJ* caused excess flipping of the PG precursor and hence produced feedback signals for *murA* and *murB* upregulation. Although RNA silencing results showed that inhibition of *ftsW/murJ* could not reduce the bulging morphology, *murA/murB* silencing significantly reduced the bulging. As previously mentioned, *murA/murB* are the common enzymes required at the beginning of the PG synthesis pathway; inhibition of these enzymes reduced the bulging morphology irrespective of FtsE overproduction. Moreover, *murJ* is dependent on *ftsW* for its localization and not for its activity (35); hence, the silencing of *ftsW* and *murJ* separately produced no significant reduction in the bulging morphology. In addition to *ftsW* and *murJ*, we also observed an upregulation in the *FtsQLB* complex from our real-time and transcriptome data. The FtsQLB complex is already known for activating FtsW and the FtsI complex for septal PG synthesis (36). In the future, the roles of FtsE and FtsQLB complex can be studied together. From the qRT-PCR and transcriptome data, we also observed a slight increase in the expression levels of other cell division proteins. Because with FtsE expression, cells grew fine without disturbing cell division, we assumed that for a proper cell division under a condition of excessive PG synthesis, increased expression of cell division proteins might be a cell survival strategy.

### Mid-cell localization of FtsE is necessary for septal PG synthesis.

It is reported that the ATPase-deficient mutant of FtsE could not rescue the chaining phenotype of Δ*ftsEX* cells, which suggests that ATPase activity of FtsE is important for FtsX-mediated activation of amidases during septal PG hydrolysis (17). However, our results showed that both the ATPase-deficient mutants, FtsE^K41A^ and FtsE^D162A^, manifested a similar bulging morphology to wild-type FtsE. When we examined cells overexpressing these mutants under a fluorescence microscope, interestingly, we observed that both FtsE^K41A^ and FtsE^D162A^ localized to the bulging site (Fig. 6A). These data showed that unlike septal PG hydrolysis, in which FtsE ATPase activity plays an important role, mid-cell localization and not the ATPase activity of FtsE is necessary for septal PG synthesis (Fig. 6A; Fig. S8A).

Neither FtsE nor FtsX alone can rescue the chaining phenotype of Δ*ftsEX* strain caused by the low-osmolarity condition, which strongly suggests that both proteins are essential for septal PG hydrolysis (16). Interestingly, the bulging morphology was observed even in Δ*FtsX* strains, which shows that FtsE alone can initiate the bulging (Fig. 6B). Because FtsE localizes just after FtsZ and interacts with it even in the absence of FtsX, it is likely that after Z ring formation, FtsE emits the signal to initiate septal PG synthesis. We also observed both a chaining phenotype and a bulging phenotype in Δ*FtsEX* strain upon FtsE overexpression, suggesting that the septal PG synthesis is independent of septal PG hydrolysis. These observations, along with previously published data, suggest that the FtsEX complex plays a role in PG hydrolysis at the mid-cell, whereas FtsE activates septal PG synthesis. Nevertheless, how FtsE activates septal PG synthesis is still unclear. Therefore, in the future, it would be interesting to determine the direct target of FtsE for the initiation of septal PG synthesis.

### Model showing the role of FtsE during PG synthesis.

**FIG 7:**
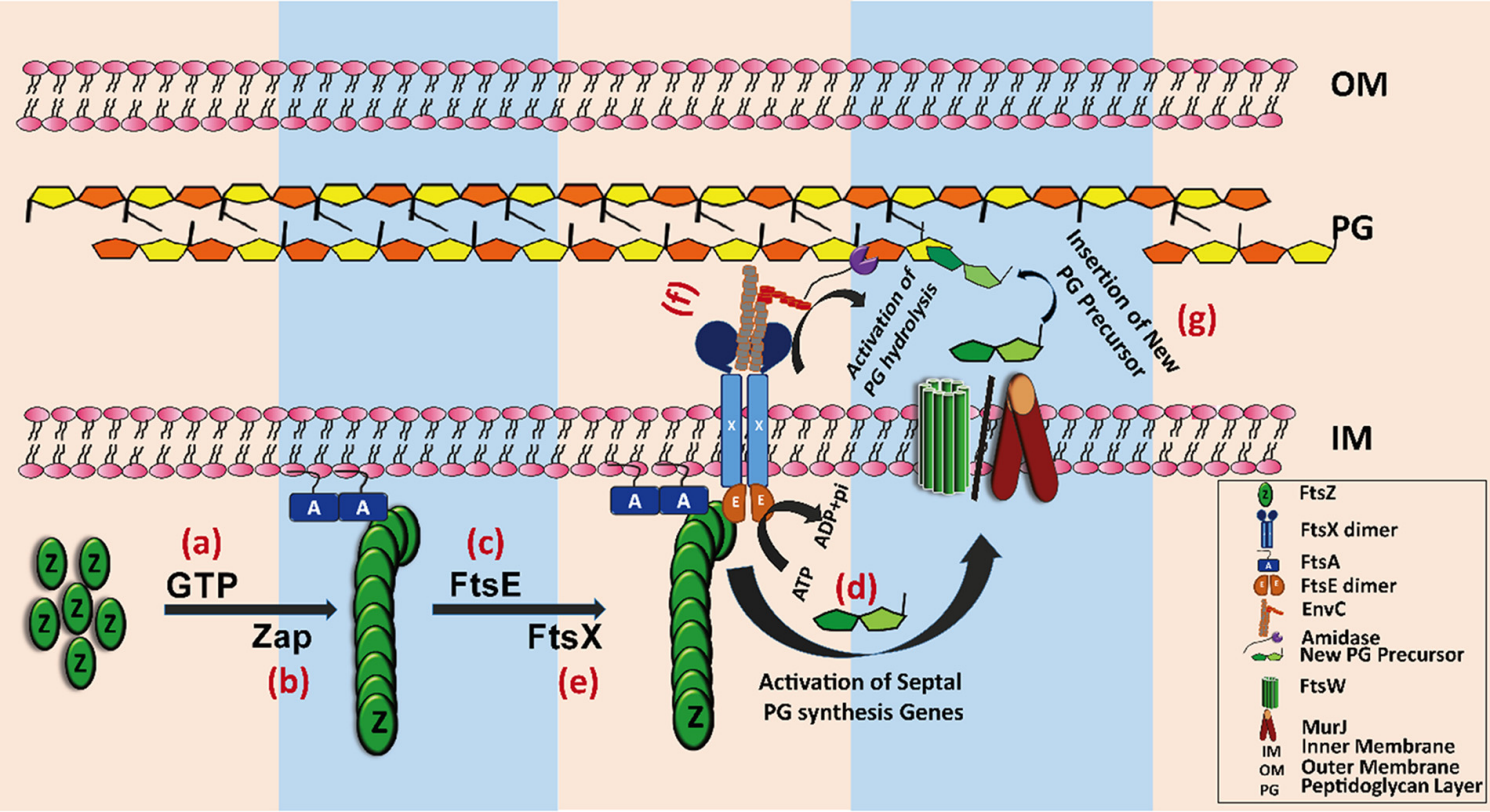
Hypothesis model. (a) FtsZ monomer polymerizes in the presence of GTP and forms short polymers. (b) Short filaments of FtsZ form bundles with the help of Zap proteins and finally a discontinuous ring at the mid-cell. This ring then anchors to the inner membrane by FtsA. (c) After the formation of ring at the mid-cell, FtsE localizes to the mid-cell and interacts with FtsZ, (d) FtsE sends the signal for septal PG synthesis. (e) After the localization of FtsE, FtsX localizes to the mid-cell and interacts with FtsE through its cytoplasmic loop; (f) then, FtsX interacts with EnvC in the periplasm through its large periplasmic loop and activates amidases A and B. Activation of amidases initiates septal PG hydrolysis. (g) After septal PG hydrolysis, the newly synthesized PG precursor flips to the periplasmic side and inserts into the old PG stand.

FtsEX complex plays a vital role during the PG layer hydrolysis and is considered one of the coordinators between the divisome complex and the septal cell wall formation (16). When cell wall hydrolysis occurs by the activation of AmiA/AmiB mutants that do not require FtsEX for their activity, cell wall hydrolysis does not coordinate with PG synthesis, and the cells are lysed. Similarly, when septal PG synthesis occurs without FtsEX-controlled cell wall hydrolysis (37), delayed cell separation results in a chaining phenotype or longer cells (16) (Fig. 6). However, very little is known about how cell constriction is coordinated with cell wall synthesis. In this study, we provide evidence that FtsE is one of the components in the divisome complex that increases PG synthesis by enhancing the expression of proteins involved in PG synthesis. Based on our data and previously published literature, we propose the following model for how FtsE coordinates membrane constriction with PG synthesis (Fig. 7). Briefly, after the Z-ring is anchored to the inner membrane, FtsE is recruited to the mid-cell through its interaction with FtsZ, and the FtsE-FtsZ interaction acts as a signal to initiate septal PG synthesis by activating PG synthesis genes. The newly synthesized PG precursors are then flipped to the periplasm with the help of flippases. Simultaneously, FtsX localizes to the mid-cell by interacting with its cytoplasmic loop with FtsE. The ATP hydrolysis by the FtsEX complex then activates amidases A and B through EnvC to initiate septal PG hydrolysis. The FtsEX ATP hydrolysis cycle controls the rate of PG hydrolysis at the mid-cell. Next, the hydrolysis of the old PG layer allows the insertion of newly synthesized PG precursors to form the septal PG layer. Thus, FtsE regulates PG synthesis and cell wall hydrolysis along with FtsX. Once the cell division is completed, the Z-ring dissociates itself from the division site. The dissociation of the Z-ring disrupts the FtsE-FtsZ interaction, which delocalizes FtsE from the mid-cell, and septal PG synthesis and hydrolysis stop.

## MATERIALS AND METHODS

### Materials.

All the chemicals, bacterial strains, and plasmids used for this study are listed in supplemental material files S1 and S2.

### Cloning and mutagenesis.

For FtsE overexpression *in vivo*, the *ftsE* ORF from E. coli K-12 genomic DNA was amplified and cloned in the pGEX-6P-1 vector between the EcoRI and SalI sites so that a GST (glutathione *S*-transferase)-tag is fused to the N terminus of FtsE. The recombinant plasmid was transformed into *DH5α* competent cells and grown on LB-Amp plates at 37°C for 14 to 16 h. The clones were confirmed by colony PCR, restriction digestion, and DNA sequencing. As described previously, point mutations for ATP mutants were generated by inverse PCR mutagenesis in wild-type *ftsE* (33) and the mutations were confirmed by DNA sequencing.

### Cell viability assay.

Cell viability analysis was done in E. coli (MG1655) cells with GST tagged-FtsE. The cells were grown until reaching an OD_600_ (optical density at 600 nm) of 0.3 to 0.4, and FtsE was overexpressed by adding different concentrations of IPTG up to 0.1 mM. After the first 1 h of FtsE induction, the cells were serially diluted with phosphate-buffered saline (PBS), and 3 of μL culture was spotted in an LB agar plate and incubated at 37°C for 12 to 16 h (26).

### Overexpression of FtsE and bacterial staining.

The GST tagged-FtsE was overexpressed in MG1655 cells by inoculating an overnight culture in fresh LB medium with 100 μg/μL of ampicillin. The culture was grown at 37°C and 200 rpm until the mid-log phase and induced with different concentrations of IPTG for 1 h. The cells were harvested and washed three times with PBS. The inner membrane staining was performed by incubating the live bacteria with FM 4-64^FX^ (1 μM) for 15 to 20 min at 37°C (26, 36). The outer membrane staining was performed by incubating the cells with Alexa Fluor 594 NHS Ester (10 μg/mL) at room temperature for 1 to 2 h (27). The nucleoid staining was performed by incubating the cells with DAPI (4′,6-diamidino-2-phenylindole; 1 μg/mL) for 5 min at room temperature (36). The cells were visualized using an Olympus fluorescence microscope (BX51).

### Fluorescent-vancomycin labeling.

FtsE-overexpressing cells were fixed with 2.8% formaldehyde at room temperature for 1 h followed by three washes with PBS. The cells were permeabilized with 0.1% Triton X-100 in PBS for 45 min at room temperature and then washed twice with PBS, followed by treatment with lysozyme (10 μg/mL) and EDTA (5 mM) for 45 min at room temperature. After permeabilization, the cells were incubated with 1 μg/μL Van-FL (29) at room temperature for 30 min and washed three times with PBS. Fluorescence imaging was performed using a fluorescence microscope.

### NADA labeling.

NADA was synthesized as described previously (30) (Fig. S9). For NADA labeling, cells were overexpressed with different concentrations of IPTG for 20 min and treated with a final concentration of 4 mM NADA. The culture was incubated at 37°C in a shaker incubator for 1 to 2 h. Before imaging, cells were washed three times with PBS and slides were prepared with a low-melting agarose pad. Imaging was done using a fluorescence microscope (Olympus BX51).

### qRT-PCR.

MG1655 *E. coli* cells with GST tagged-FtsE were overexpressed with different concentrations of IPTG for 1 h and harvested at 2,500 × *g* for 10 min. RNA was isolated from the overexpressed cells using a HiMedia RNA isolation kit, and cDNA synthesis was carried out with an equal amount of RNA (~1 to 2 μg). qRT-PCR was performed with Roche/Applied Biosystem-SYBR Green using a StepOne Plus real-time PCR machine. The analysis was performed using the 16S/GAPDH (glyceraldehyde-3-phosphate dehydrogenase) gene as an internal control.

### Scanning electron microscopy.

For scanning electron microscopy, FtsE-overexpressing cells were harvested at 2,500 × *g* for 10 min and fixed with 2.8% formaldehyde and 0.04% glutaraldehyde in PBS at room temperature for 1 h. The cells were washed three times with water, placed on a poly l-lysine-coated coverslip, and incubated for 1 h at room temperature. The cells bound to the coverslip were dehydrated with an increasing gradient of ethanol in water (30% to 100%) at room temperature and then dried at 37°C overnight. Imaging was carried out with a scanning electron microscope (Carl Zeiss Evo18).

### FtsZ monoclonal antibody production.

To obtain the FtsZ monoclonal antibody, FtsZ protein was purified by following glutamate based protocol (38). The purified protein was given to Abgenex Private Ltd., Bhubaneswar for rearing antibodies in mice.

### Immunofluorescence microscopy.

Bacterial culture after FtsE overexpression was harvested by centrifugation at 2,500 × *g* for 10 min, fixed with fixing reagents (2.8% formaldehyde and 0.04% glutaraldehyde in PBS), and incubated at room temperature for 15 to 20 min, followed by two washes with PBS. The cells were permeabilized with 0.1% Triton X-100 in PBS for 45 min at room temperature and washed twice with PBS. This process was continued by incubating the cells with 10 μg/mL of lysozyme and 5 mM EDTA in PBS for 45 min at room temperature. This followed by two washes with PBS. Blocking was performed with blocking buffer (1% BSA in PBS) for 30 min at 37°C. After blocking, cells were incubated overnight with the primary antibody (anti-mouse FtsZ at a 1:200 ratio and anti-mouse His at 1:10,000) in the same blocking buffer at 4°C. The next day, cells were washed twice with 0.05% Tween 20 in PBS (PBST), incubated with the secondary antibody (mouse/rabbit anti-goat conjugated with Alexa-488) in the blocking buffer at 37°C for 1 to 2 h, and washed twice with PBST. The cells were stained with DAPI at a final concentration of 1 μg/mL at room temperature for 5 min and washed with PBS. The slides were prepared with a 1% low-melting agarose pad and images were taken with an Olympus fluorescence microscope. For membrane staining, FM 4-64^FX^ was used at a final concentration of 1 μM.

### Time-lapse experiment.

For the time-lapse experiment, FtsE-overexpressing MG1655 cells were grown until reaching an OD_600_ of 0.3 to 0.4. An agarose pad (0.5%) for visualization was prepared in a concavity slide with the required concentration of IPTG (22). The slide was incubated at 37°C for 10 min. The time series experiment was performed using the Cell Discover 7 microscope (Zeiss) at 37°C for 2 to 4 h. Images were taken at 3-min intervals and a movie was made using Windows Live Movie Maker.

### Live-dead assay.

After overexpression of GST tagged-FtsE for 1 h in MG1655 cells, 1 mL of culture was taken and harvested by centrifugation at 10,000 × *g* for 10 min. Cells were washed with 0.85% NaCl and incubated at room temperature for 45 min. The cells were then centrifuged and treated with SYTO 9 and PI at an equal ratio in 0.85% NaCl at room temperature for 30 min. Cells were washed twice with water and visualized by fluorescence microscopy.

### Transcriptome analysis.

Transcriptome analysis was performed with the help of Genotypic (Bengaluru, India). After FtsE overexpression, cells were washed three times with pre-chilled PBS buffer and stored at −80°C after flash-freezing in liquid nitrogen. RNA was isolated, and a cDNA library was prepared using the NEBNext Ultra Directional RNA Library Prep kit. mRNA sequencing was performed using the NextSeq 150-paired-end method. Next, reference-based analysis was carried out, and the fold change for each gene was calculated following the formula provided by the service provider, using the read-counts.

### Western blotting.

For Western blot analysis, cells were harvested and resuspended with equal amounts of a mixture of water and 6× SDS loading dye, run on SDS-PAGE, and transferred to an activated PVDF (polyvinylidene difluoride) membrane. The blot was incubated with the required primary antibody at 4°C overnight, washed, and treated with the corresponding secondary antibody at room temperature for 1 h. The blot was developed using Pierce ECL Plus Western Blotting Substrate.

### Expressed antisense assay.

Antisense RNAs for each gene were expressed using previously published protocols (33). The plasmid *pHN678* was a kind gift from Liam Good. Oligonucleotides for the cloning were synthesized according to published procedures. Because the antisense construct is under the control of T7 promoter (IPTG-controlled), to co-transform FtsE, we generated an *ftsE/pBADHis* construct which was under arabinose control. To express antisense RNA, *ftsE/pBADHis* and *murA-pHN678 (murB*, *ftsW*, and *murJ)* were co-transformed into E. coli BL21 competent cells. The cells were grown until reaching an OD_600_ of 0.4 to 0.5 and induced with 0.1% arabinose and different concentrations of IPTG (50 to 250 μM) simultaneously for 4 h at 37°C. The cells were harvested and washed twice with PBS. Cells were fixed and permeabilized using the same protocol that was used for immunostaining. The cells were then stained with Van-FL for 30 min at room temperature, followed by two washes with PBS. Imaging was done on an agarose pad using an ApoTome.2 (Zeiss) fluorescence microscope.
