# Supplementary material for: FtsE, the Nucleotide Binding Domain of the ABC Transporter Homolog FtsEX, Regulates Septal PG Synthesis in E. coli
Source: Microbiol Spectr. 2023 Apr 4;11(3):e02863-22. doi: 10.1128/spectrum.02863-22 (PMC10269673; doi:10.1128/spectrum.02863-22)
Supplement: Supplemental file 2 — Supplemental material. Download spectrum.02863-22-s0001.pdf, PDF file, 2.5 MB [file spectrum.02863-22-s0001.pdf]

# Supplementary Data

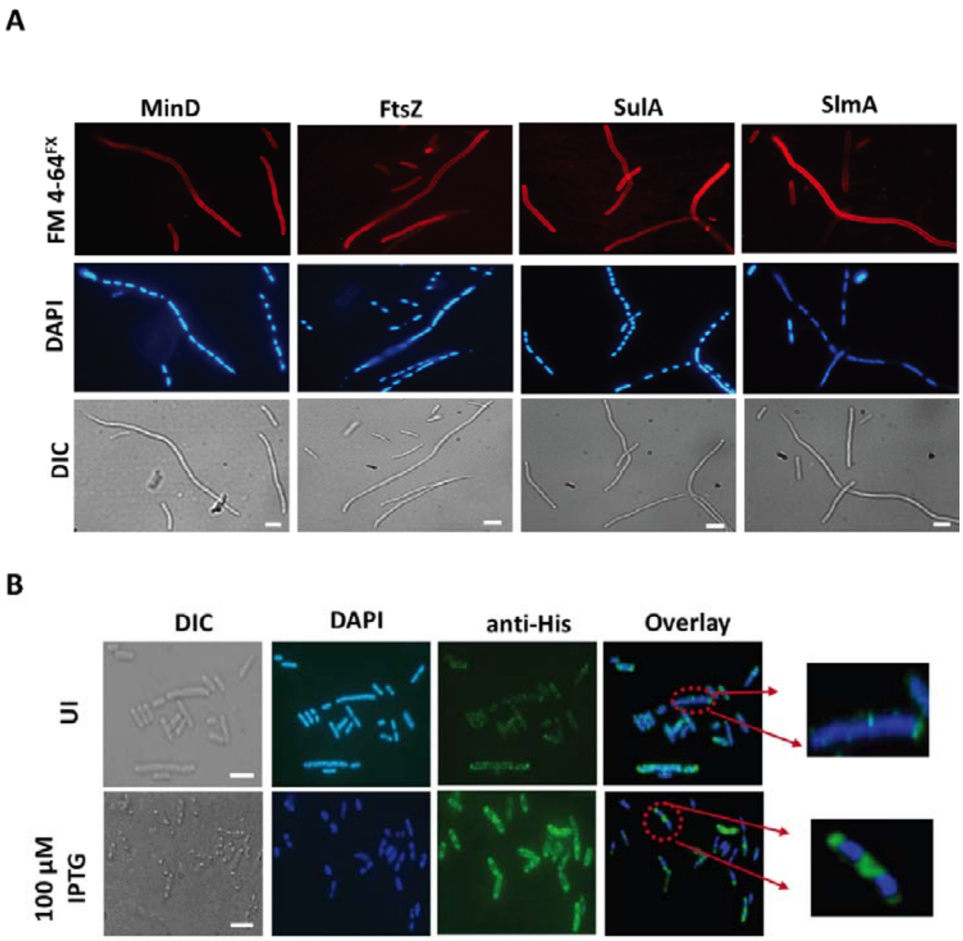


***Figure S1. Bulging morphology of E. coli upon protein overexpression is specific to FtsE. Panel A*** shows the overexpression of different cell division proteins (FtsZ, SlmA, SulA and MinD), leading to formation of smooth filaments. ***Panel B,*** shows the immunofluorescence images of FtsE overexpressed cells, Lane 1 - DIC, Lane 2 - Nucleoids (Blue), Lane 3 - His-FtsE (Green). (Scale Bar 4 µm)

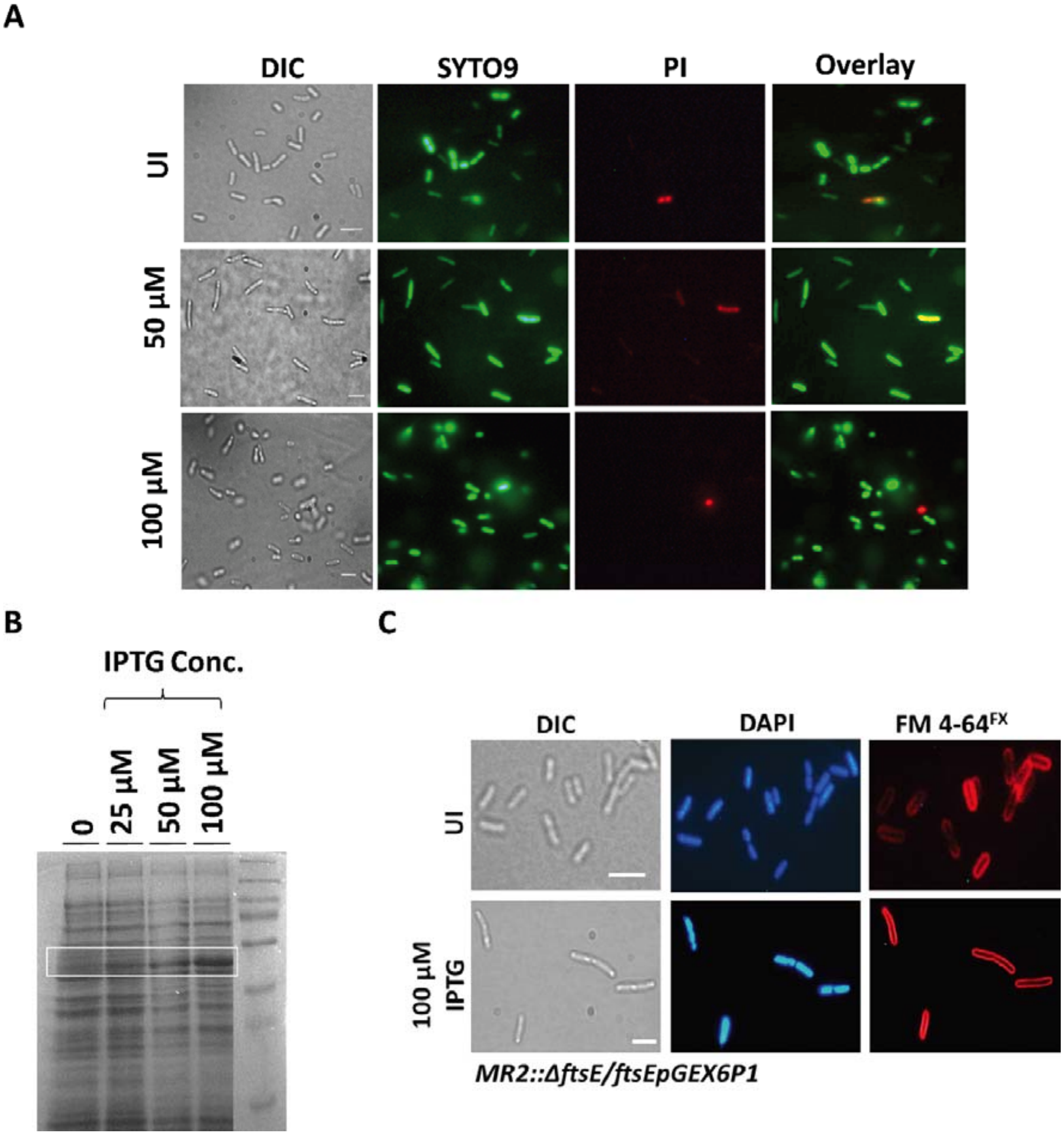


***Figure S2. Overexpression of FtsE in not toxic to E. coli. Panel A*** **Shows** Live-Dead assay after overexpression of FtsE with 50 and 100 µM IPTG: (Lane 1, DIC images), (Lane 2, live cells in green), (Lane 3, dead cells in red) and (Lane 4, overlay of Lane 2 & 3). ***Panel B*** is showing FtsE protein level in different concentration of IPTG. Scale Bar 4 µm. ***Panel C*** His-FtsE was overexpressed in FtsE deletion background (*MR2::ΔftsE: Kan).* Lane 1, DIC images, Lane 2, Nucleoids (Blue) and Lane 3, inner membrane (Red). (Scale Bar 4 µm)

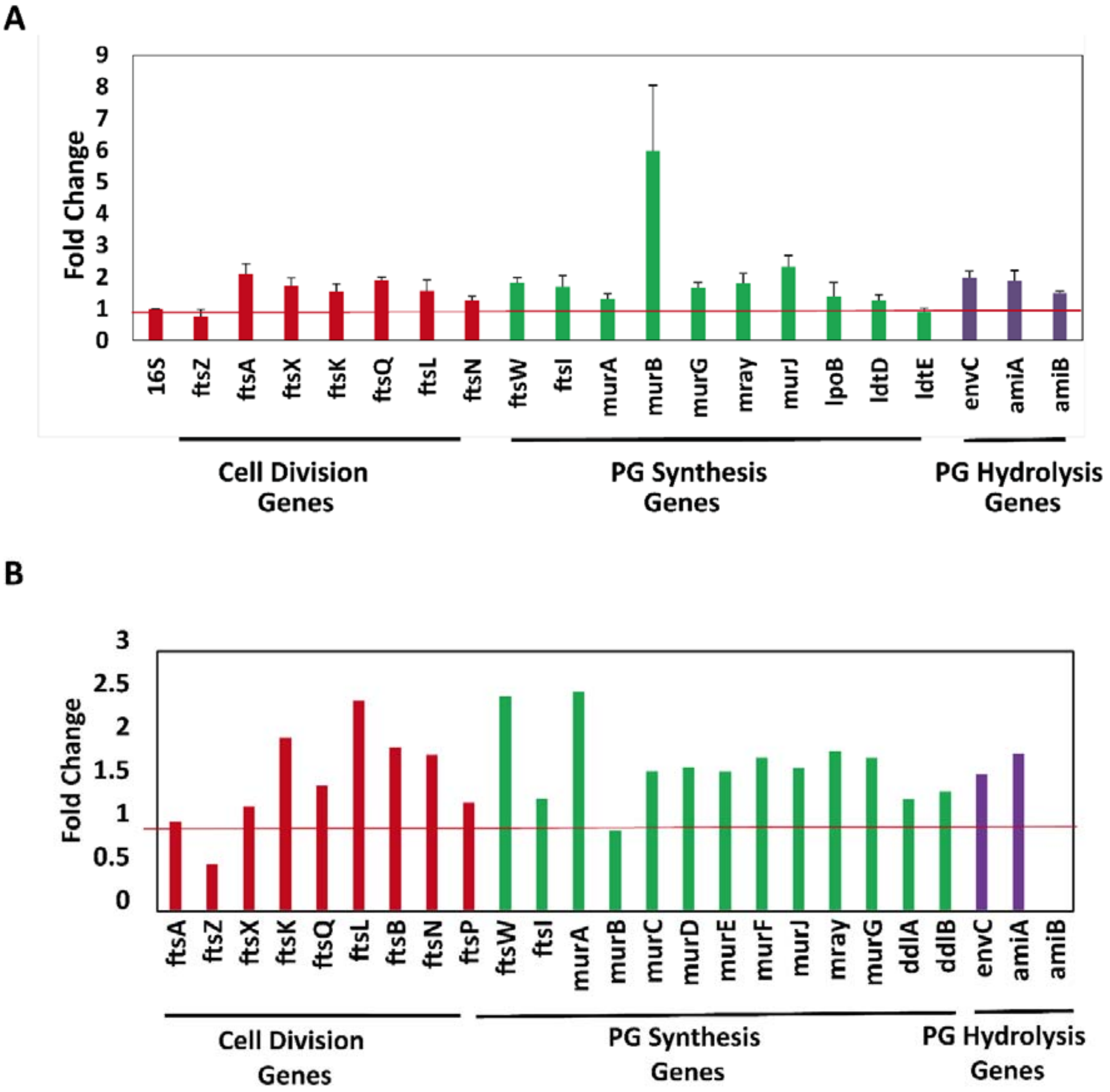


***Figure S3. Gene regulation in FtsE overexpressed E. coli. Panel A*** Real-time PCR data showing gene regulation in FtsE overexpressed MG1655. ***Panel B*** Transcriptome-profiling of genes upon FtsE overexpression in MG1655.

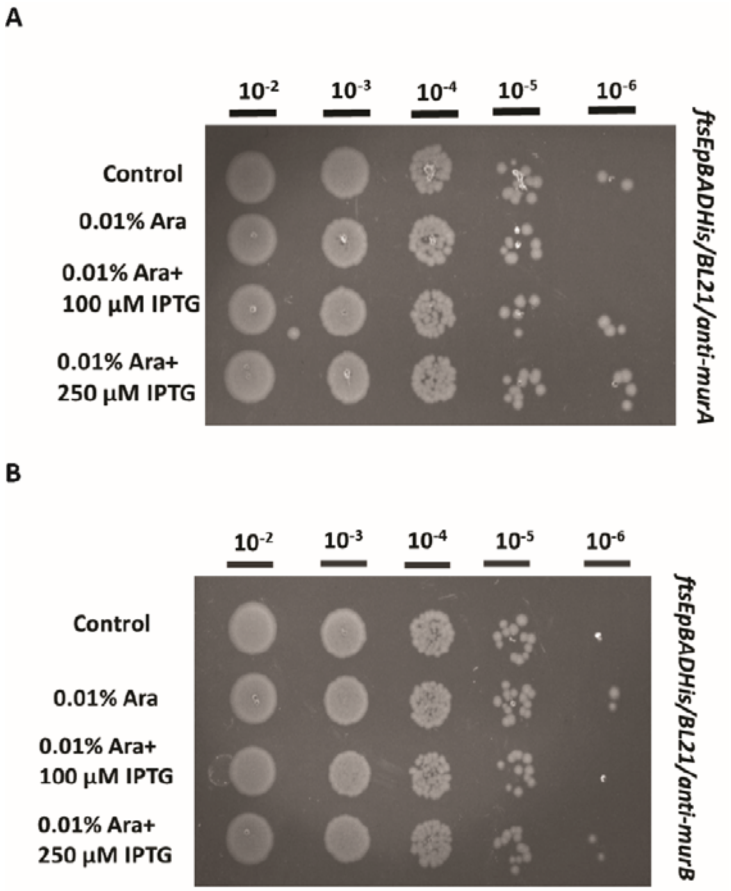


***Figure S4. Cell viability assay for anti-sense inhibition in FtsE overexpressed cell. Panel A*** shows cell viability upon *murA* inhibition in FtsE overexpressed cells. ***Panel B*** shows cell viability upon *murB* inhibition in FtsE overexpressed cells.

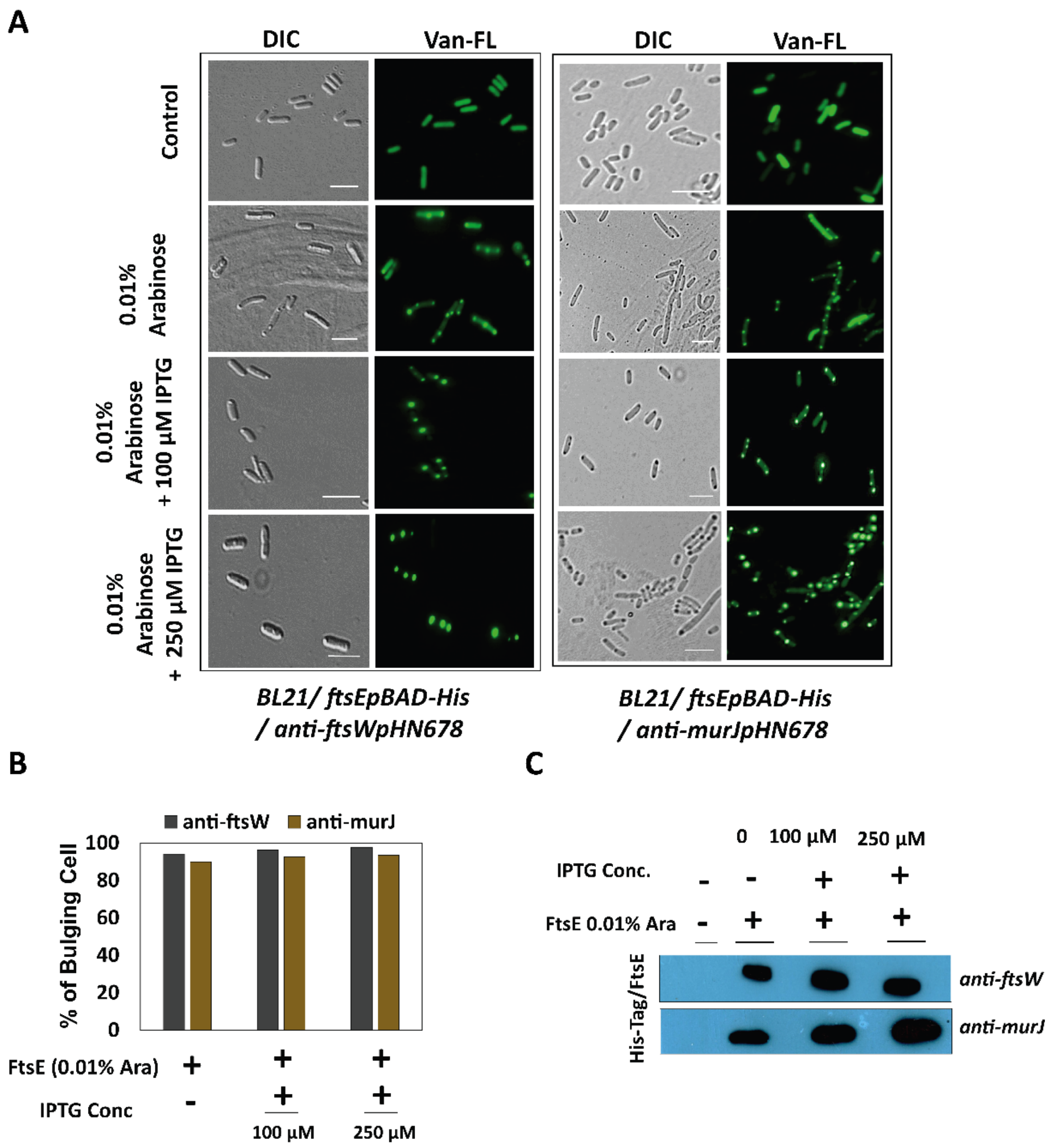


***Figure S5. Silencing of flippase (FtsW and MurJ) do not supress the bulging morphology. Panel A*** shows reduction in bulging morphology upon *ftsW (left side)* and *murJ (right side)* silencing. Fluorescent vancomycin (Van-FL) labeling was done to see the reduction. ***Panel B*** shows the percentage of cells with bulging phenotype, where (*ftsW*) control n=167, *anti-ftsW* 100 µM IPTG n=289, *anti-ftsW* 250 µM IPTG n=300 and (*murJ)* control n=305, *anti-murJ* 100 µM IPTG n=282, *anti-murJ* 250 µM IPTG n=258. ***Panel C*** shows presence of FtsE protein in cell, where *ftsW* and *murJ* were silenced. (Scale Bar 5 µm).

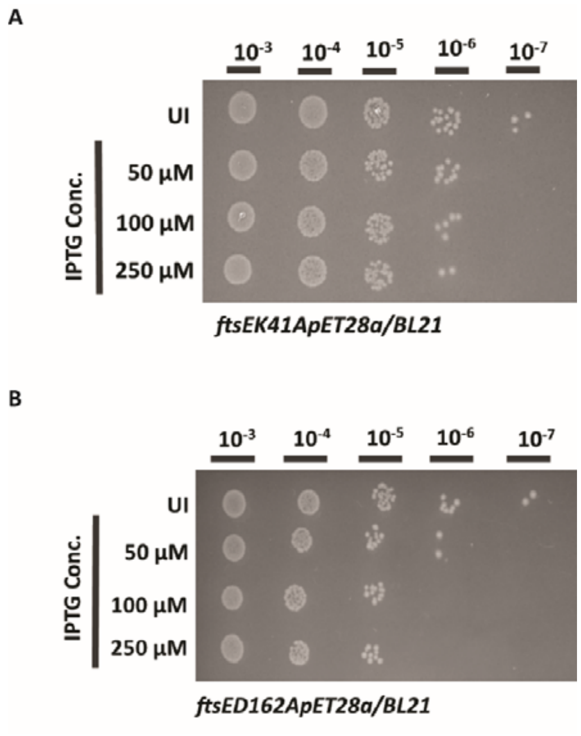


***Figure S6. Cell viability assay for FtsE ATP mutants upon overexpression in MG1655.*** ***Panel A*** *ftsEK41A* and ***Panel B*** *ftsED162A*

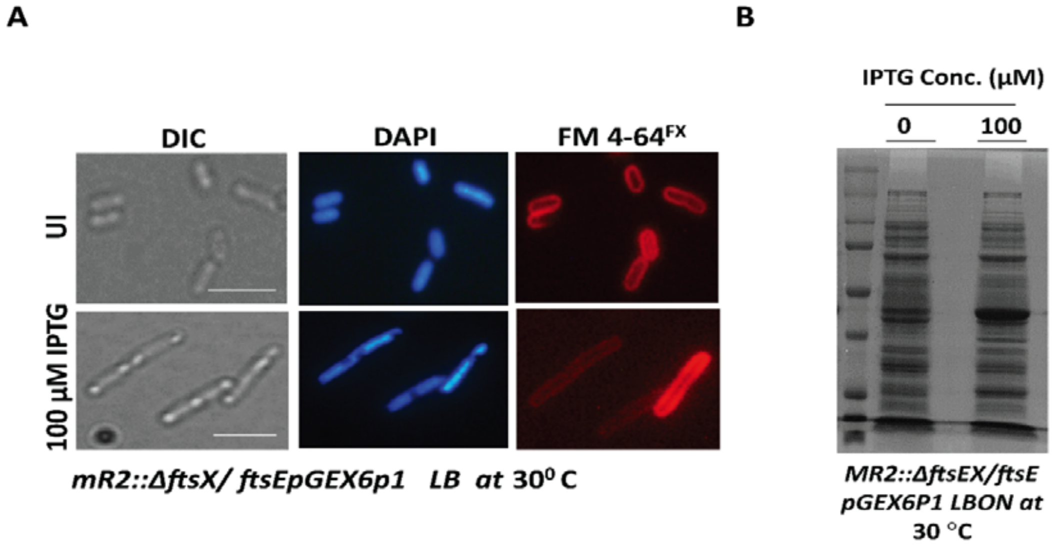


***Figure S7*. Overexpression of FtsE in absence of FtsX.** ***Panel A*** shows bulging morphology by FtsE overexpression in the absence of FtsX (Scale Bar 4 µm). ***Panel B*** shows the level of FtsE protein after overexpression in SDS PAGE in FtsEX deletion strain in LBON media.

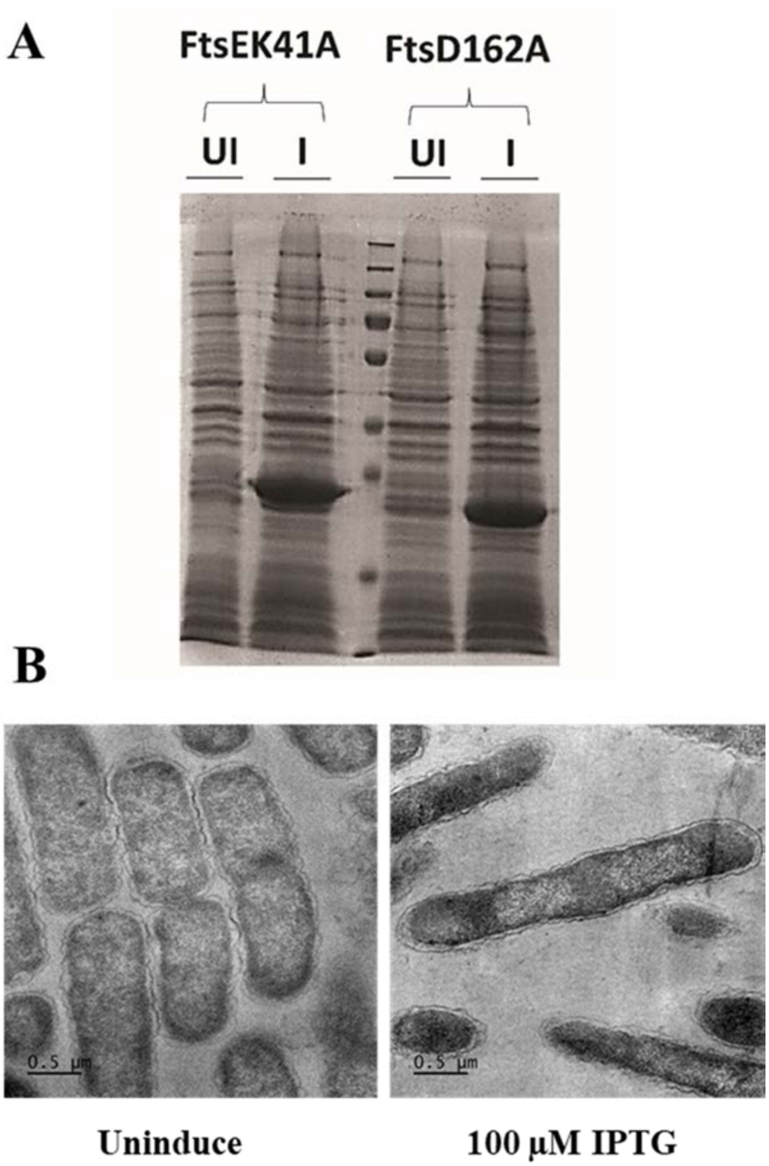


***Figure S8. Overexpression of FtsE ATP mutant in Wildtype E.coli. Panel A*** Shows the Protein level of all the ATP mutant in SDS PAGE in wildtype *E.coli*. ***Panel B*** shows bulging through TEM images upon FtsE overexpression.

**A**

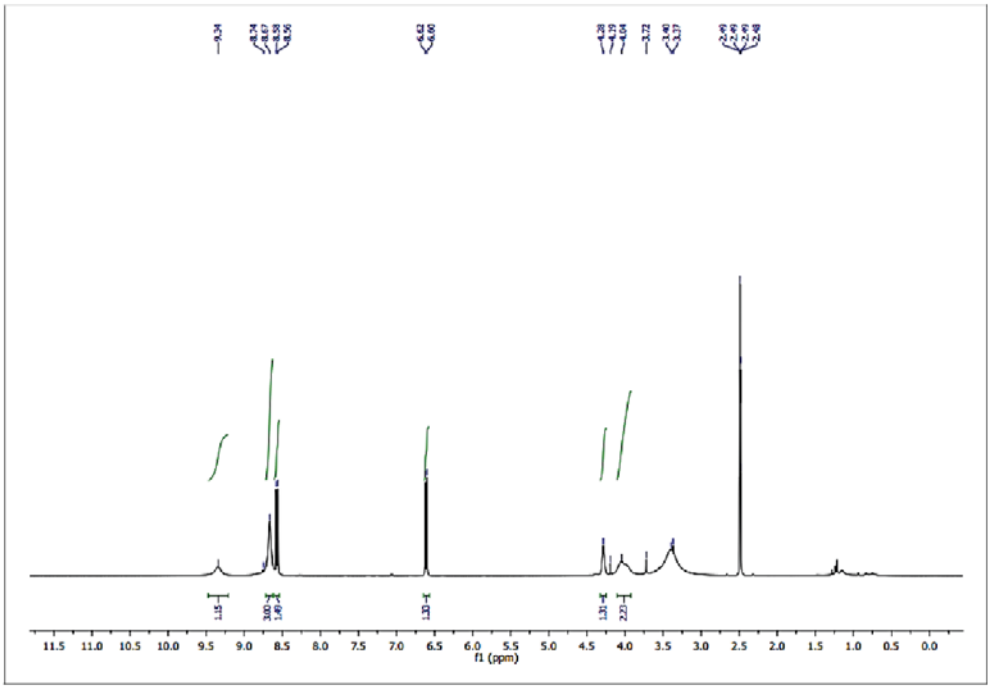


**B**

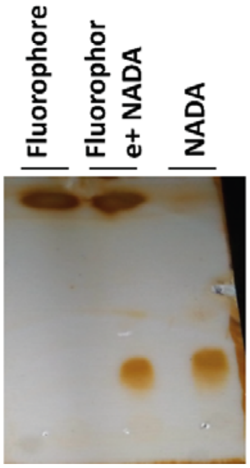


***Figure S9. Purity and confirmation of NADA. Panel A*** shows the TLC images of the synthesized NADA molecule. ***Panel B*** shows the NMR data of the NADA molecule.

**Table.1**

| Strain or Plasmid | Relevant Feature(s) | Construction, Source, or References |
|---|---|---|
| **Strain** | | |
| MG1655 | Wildtype | Laboratory Collection |
| DH5α | | Laboratory Collection |
| BL21 | | Laboratory Collection |
| *ΔftsE* | mR2 *ΔftsE:: Kan* | Reddy 2007 |
| *ΔftsX* | mR2 *ΔftsX:: Kan* | Reddy 2007 |
| *ΔftsEX* | mR2 *ΔftsEX:: Kan* | Reddy 2007 |
| | mR2*::ΔftsX/ ftsEpGEX6p1* | In this study |
| | mR2*::ΔftsEX/ftsEpGEX6p1* | In this study |
| | *BL21/ ftsEpBAD-His/ anti-ftsWpHN678* | In this study |
| | *BL21/ ftsEpBAD-His/ anti-murJpHN678* | In this study |
| | *BL21/ftsEpBAD-His/* anti-*murApHN678* | In this study |
| | *MG1655/ftsEpGEX6P1* | In this study |
| **Plasmid** | | |
| pGEX6P1 | *tac promoter, Amp, GST C-terminal tag* | Laboratory Collection |
| pBADHis | *pBAD18, ara promoter, Amp ,His N-terminal tag* | In this study |
| pHN678 | *trc promoter, Chl, expressed RNAi* | Goh et al. 2009 |
| pGEXE1 | *pGEX6P1-FtsE* | In this study |
| pBADE1 | *pBAD18His-FtsE* | In this study |
| pHNmurA (anti-murA) | *pHN678-murA, antisense* | In this study |
| pHNmurB (anti-murB) | *pHN678-murB, antisense* | In this study |
| pHNftsW (anti-ftsW) | *pHN678-ftsW, antisense* | In this study |
| pHNmurJ (anti-murJ) | *pHN678-murJ, antisense* | In this study |
| pGEXEK41A | *pGEX6P1-FtsEK41A* | In this study |
| pGEXED162A | *pGEX6P1-FtsED162A* | In this study |

***ST1***. List of plasmids and strain used during the whole stud**y**.

**Table.2**

| Reagents | Company | References |
|---|---|---|
| **Media:** | | |
| LB-Broth | Himedia, India | |
| LB-Agar | Himedia, India | |
| **Chemicals:** | | |
| FM 4-64 (FX) | Invitrogen | *Panda et al. 2015* |
| DAPI | Sigma | |
| Poly-L-lysine | Sigma | |
| His-Monoclonal antibody | Sigma | |
| Secondary anti-mouse and anti-rabbit antibody | Invitrogen | |
| ECL Plus Western blot solution | Invitrogen | |
| SYBR Green Real time Master Mix | Roche and Applied Biosystem | |
| Alexa Fluor™594 NHS Ester | Invitrogen | *Priyadarshini et al, 2007* |
| Live-Dead Assay Kit | Invitrogen | |
| FtsZ Monoclonal antibody | Customized, Abagenex, India | |
| NADA (Synthesized) | IIT, Bhubaneswar, Odisha | *Erkin Kuru et al, 2014* |
| Vancomycin, BODIPY™ FL Conjugate (BODIPY™ FL Vancomycin) | Invitrogen | *Walker et al, 2006* |

***ST2***. List of media and chemicals used during the whole study

***SV1.*** Video showing bulging morphology initiates at mid-cell and then moves to the pole
